# Investigating the Neural Correlates of a Streaming Percept in an Informational-Masking Paradigm

**DOI:** 10.1371/journal.pone.0114427

**Published:** 2014-12-09

**Authors:** Sahar Akram, Bernhard Englitz, Mounya Elhilali, Jonathan Z. Simon, Shihab A. Shamma

**Affiliations:** 1 The Institute for Systems Research, University of Maryland, College Park, Maryland, United States of America; 2 Department of Electrical and Computer Engineering, University of Maryland, College Park, Maryland, United States of America; 3 Department of Biology, University of Maryland University, College Park, Maryland, United States of America; 4 Department of Electrical and Computer Engineering, Johns Hopkins University, Baltimore, Maryland, United States of America; 5 Département d'Etudes Cognitives, Ecole normale supérieure, Paris, France; 6 Department of Neurophysiology, Donders Institute for Brain, Cognition and Behaviour, Nijmegen, The Netherlands; UNLV, United States of America

## Abstract

Humans routinely segregate a complex acoustic scene into different auditory streams, through the extraction of bottom-up perceptual cues and the use of top-down selective attention. To determine the neural mechanisms underlying this process, neural responses obtained through magnetoencephalography (MEG) were correlated with behavioral performance in the context of an informational masking paradigm. In half the trials, subjects were asked to detect frequency deviants in a target stream, consisting of a rhythmic tone sequence, embedded in a separate masker stream composed of a random cloud of tones. In the other half of the trials, subjects were exposed to identical stimuli but asked to perform a different task—to detect tone-length changes in the random cloud of tones. In order to verify that the normalized neural response to the target sequence served as an indicator of streaming, we correlated neural responses with behavioral performance under a variety of stimulus parameters (target tone rate, target tone frequency, and the “protection zone”, that is, the spectral area with no tones around the target frequency) and attentional states (changing task objective while maintaining the same stimuli). In all conditions that facilitated target/masker streaming behaviorally, MEG normalized neural responses also changed in a manner consistent with the behavior. Thus, attending to the target stream caused a significant increase in power and phase coherence of the responses in recording channels correlated with an increase in the behavioral performance of the listeners. Normalized neural target responses also increased as the protection zone widened and as the frequency of the target tones increased. Finally, when the target sequence rate increased, the buildup of the normalized neural responses was significantly faster, mirroring the accelerated buildup of the streaming percepts. Our data thus support close links between the perceptual and neural consequences of the auditory stream segregation.

## Introduction

The segregation of an auditory scene into multiple streams is a highly complex task facilitated by informative cues in the acoustic stimulus along both the temporal and spectral dimensions. Although there have been intensive studies on the behavioral and neural bases of auditory stream segregation over the last decades, key features of this process still remain to be explored [1]–[3].

**Figure 1:**
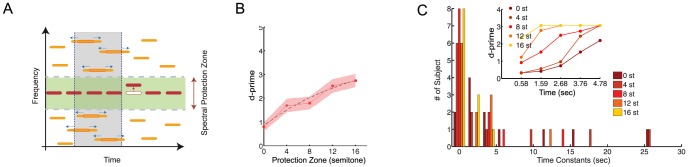
Stimulus paradigm and behavioral performance. (A) Schematic representation of the stimulus design. A rhythmic sequence of pure tones (target sequence, red) is placed within a background of randomly distributed (in time and frequency) tones (maskers, yellow) and ‘protected’ by a spectral zone with no stimulus energy (green region). In the target task, subjects detected a randomly occurring frequency-shifted tone (red arrow). In the masker task, subjects were instructed to detect an elongation of all constituent tones of the masker in a 0.5 s time window (blue arrows). Each trial contained only one type of deviant, both, or none. Subjects performed the tasks in separate blocks, with the order counterbalanced across subjects. (B) Behavioral performance in the target task as a function of protection zone in a range from 0 to 16 semitones (Psychoacoustic experiment A, N = 14) (C) Behavioral build-up of detection in the target task. Histogram of time constants obtained from exponential fitting to the buildup curves of the behavioral responses as a function of the size of the protection zone (0 to16 semitones). The inset shows behavioral buildup of target task detection for a sample subject illustrating the changes in the buildup speed as a function of different protection zone sizes.

A commonly used paradigm for studying auditory perceptual organization is paired sequences of pure tones, alternating in time, which can be perceived as either a single or two segregated auditory objects, under different conditions [1], [4]. Such tone sequences have been instrumental in unraveling a number of perceptual processes and neural underpinning underlying streaming, but these stimuli are limited in how closely they reflect realistic auditory streams occurring in everyday environments. It has been proposed that use of a spectrotemporally richer stimulus commonly used for “informational masking” (IM) studies [5] invokes similar mechanisms, but also provides many more degrees of freedom to probe additional mechanism involved in streaming. Such a stimulus typically consists of a target tone sequence embedded in a cloud of masker tones that are randomly desynchronized (Fig. 1A) and has been shown to yield streaming percepts analogous to those of the simpler two-tone sequences [5]–[8], both in their systematic dependence on stimulus parameters, as well as the improvement of detection over the time course of few seconds (the so-called buildup of streaming [9]–[11]).

As in an earlier study [12], we expanded the investigation of the premise that such informational masking stimuli invoke similar mechanisms to those involved in steam segregation. We probe the correlation between neural responses to an IM stimulus recorded using Magnetoencephalography (MEG) and behavioral responses in psychoacoustic experiments while manipulating stimulus parameters under different attentional states of the listeners. Specifically, the IM paradigm is used to explore stream formation in a single tone sequence (target) as (1) a function of target/masker separation, (2) target tone frequency and (3) target repetition rates, all while manipulating the attentional state of listeners to and away from the target sequence. In the target task, subjects detect a frequency-shifted deviant in the target sequence; in the masker task subjects detect a sudden elongation of the masker tones in time. Both tasks require focused attention, but to spectrally and temporally different features of the auditory scene. Since the stimuli presented in both tasks are identical, any difference in neural representation of the sounds is deemed a result of attentional modulation.

Overall, the results demonstrate that performance measures and neural responses to IM stimuli are correlated across all stimulus and task conditions, suggesting that the neural signal can be viewed as an indicator of the streaming percept.

A second critical goal of the current study is to investigate the different roles of different rates of temporal modulations. Modulation rates in the range of 2–10 Hz are crucially important in grouping the physical and perceptual cues in a complex acoustic scene and stream formation [13]–[15]; however, among the primarily unknown facts is the exact role of these modulatory rates and their relative importance in the streaming process.

In a study by Xiang et al. 2010 [16], the interaction between task-driven and stimulus-driven attentional processes for two competing rhythmic sequences at two relatively different rates (4 and 7 Hz) was explored. The faster sequence (7 Hz) was behaviorally quite different from the slower (4 Hz), especially for the buildup over the time course of each trial. Here, we were interested in using a paradigm based on that of [12], in which an informational masking stimulus with a repeating target note at 4 Hz in the midst of random interferers was employed. Here, though we replaced the slow target rate (4 Hz) with a faster rate (7 Hz) to explore the neural and behavioral responses to the faster presentation rate more independently as well as conducting a richer behavioral study on the buildup of target detectability as a function of target sequence presentation rate.

## Methods

The effect of manipulating stimulus parameters and attentional modulation on both task performance and neural responses was explored in 4 different experimental sections, 2 psychoacoustic (experiments A, B), and 2 MEG (experiments C, D). Psychoacoustic experiment A and MEG experiment C, investigated the effect of different spectral protection zone widths. Psychoacoustic experiment B, investigated the effect of different target sequence rates with a fixed protection zone. MEG experiment D investigated the dependence of the responses on target frequency, as well as changes in buildup and lateralization in the different tasks, all in the context of a fixed protection zone and target tone rate.

### Participants

A total of 14 subjects participated in psychoacoustic experiment A (6 male/8 female; mean age, 26 years; range 19–33 years), and 12 subjects (5 male/7 female; mean age 25 years; range 19–30 years) participated in psychoacoustic experiment B. For MEG experiment C, 12 (7 male/5 female; mean age, 25 years, range 18–33 years) participated in the study; in MEG experiment D, 12 subjects (6 male/6 female; mean age, 23 years, range 18–33 years) participated. Six subjects took part in all experiments. Psychoacoustic and MEG experiments were conducted over a period exceeding 15 months and subjects participating in different experiments were partly non-overlapping.

Participants were all right handed [17], and had no history of hearing problems or neurological disorders. Subjects were compensated for their participation. The University of Maryland Institutional Review Board approved the experiments, and written informed consent was obtained from each participant.

### Stimulus Design

The stimulus paradigm is related to previous stream segregation experiments in terms of stimulus parameters governing performance [10], [18]–[20], but using an Informational Masking stimulus, a regular foreground embedded within an irregular background, as in [12]. Specifically, each stimulus consisted of two concurrent streams; a narrow-band temporally regular target tone sequence and a wide-band cloud of tones that were temporally irregular—the masker stimulus. Subjects were asked to detect either a deviation in frequency of one tone in the target sequence (target task), or a deviation in duration in one of the masker tones (masker task), in separate blocks. Identical stimuli were thus presented in the two different tasks, but with the subject's attention guided to different aspects of the stimuli in the two tasks.

The target sequence was a sequence of identical pure tones with frequency chosen randomly in the range of 250–500 Hz (in 2 semitone steps). The pure tones were presented at a fixed rate in the range of 2–10 Hz, depending on the experimental condition. The masker stimulus formed a complex acoustic background consisting of pure tones placed at randomized temporal and spectral positions. The temporal positions of the tones were uniformly distributed over time at a density of 50 tones/s and over frequency at a spectral resolution of 2 semitones. The spectral positions were chosen uniformly in logarithmic frequency in a range of 5 octaves centered at 353 Hz (ranging from approximately 62 Hz to 1997 Hz), excluding a spectral protection zone, i.e. a frequency band around the target sequence with no masker tone allowed in it. The random sampling of frequencies kept the probability of harmonically related maskers at a minimum. The protection zone on either side of the target sequence varied in width, ranging from 0–16 semitones in 4 semitone steps. The duration of the target and masker tones was 75 ms with 10 ms onset and offset cosine ramps.

For the target and masker tasks, deviations were introduced at a randomly chosen constituent tone for either sequence, introducing a frequency, or a duration change, respectively. There were 4 types of trials: (i) null condition (no deviant); (ii) target condition (one target deviant per stimulus); (iii) masker condition (one masker deviant per stimulus); and (iv) combined condition (one target and one masker deviant independently, per stimulus). A target deviant was an upward or downward displacement, of a randomly chosen target note, from the target frequency by 2 semitones. A masker deviant was a single 500 ms window in which all masker tones starting in this window were elongated from 75 ms to 400 ms. For each condition 15 exemplars were generated, differing in the position/tone which was modified.

The stimuli were generated using MATLAB (The MathWorks). Each trial stimulus was 5.5 s long and sampled at 44.1 kHz.

### Psychoacoustic studies

Participants performed the tasks at a computer in a soundproof room. They were asked to control the computer using a Graphical User Interface (GUI) and they were allowed to adjust the volume to a comfortable level before starting the experiment. No change of stimulus intensity was allowed after starting the experiment. A complete explanation of the required task, as well as the basic instructions on using the GUI, was given in advance.

#### Psychoacoustic Experiment A

In psychoacoustic experiment A, the effect of different protection zone widths (0, 4, 8, 12 and 16 semitones) with a fixed target rate of 7 Hz was examined. A block of 200 stimuli consisting of 5 protection zones ×4 conditions ×10 exemplars were presented to the subjects. Participants could proceed from one trial to the next by pressing a button when they were ready. For this experiment, participants were required to do the target task only.

A training block of 15 trials in a decreasing order with respect to the protection zones was played for the subjects prior to the actual experiment. Participants could listen to each sound as many times as desired and after each trial, they were asked about the presence of deviants in that trial with the correct answer displayed on the screen afterwards.

For the real experiment stimulus was presented only once, and no feedback was given after each trial. This part lasted approximately 1 h.

#### Psychoacoustic Experiment B

In psychoacoustic experiment B, the protection zone was fixed to 8 semitones and the rates were varied from 2–10 Hz in steps of 2 Hz. A block of 200 trials consisted of 5 target sequence rates ×4 conditions ×10 exemplars with fixed protection zone were presented to the subjects. For each block participants were required to do the target task only.

Training sets of 20 stimuli were provided for each section and they were allowed to listen to each sound as many times as they needed to be able to perform the task. The training block was presented with rates increasing from 2 to 10 in steps of 2 Hz.

For the real experiment, stimulus was presented only once, and no feedback was given after each trial. This part lasted approximately 1 h.

Participants performed experiments A and B of the psychoacoustic experiment on 2 different days.

### MEG recordings

#### MEG Experiments C & D

The *presentation* software package (Neurobehavioral Systems) was used to present stimuli to the subjects. The sounds (approximately 70 dB SPL) were delivered to the participants' ears with 50Ω sound tubing (E-A-RTONE 3A; Etymotic Research), attached to E-A-RLINK foam plugs inserted into the ear canal. The entire acoustic delivery system is equalized to give an approximately flat transfer function from 40–3000 Hz, i.e. encompassing the range of the presently delivered stimuli.

MEG signals were recorded in a dimly lit magnetically shielded room (Yokogawa Electric Corporation) using a 160-channel whole-head system (Kanazawa Institute of Technology, Kanazawa, Japan). Its detection coils are arranged in a uniform array on a helmet-shaped surface on the bottom of the dewar, with ∼25 mm between the centers of two adjacent 15.5-mm-diameter coils. Sensors are configured as first-order axial gradiometers with a baseline of 50 mm; their field sensitivities are 5 fT/Hz or better in the white noise region.

Three of the 160 channels are magnetometers separated from the others and used as reference channels in noise-filtering methods [21]. The magnetic signals were filtered to the range of 1 Hz and 200 Hz, notch filtered at 60 Hz, and sampled at 1 kHz.

A pre-experiment consisting of 200 repetitions of a 1 kHz, 50 ms tone pip was presented before starting the real experiment. The inter-trial intervals were randomized between 0.75 ms and 1.55 s, and participants were asked to count the tone pips. The experiment was done as a control condition to check the M100 response (a prominent peak approximately 100 ms after pip onset) and verify that the location and strength of neural signals fell within a normal range.

A training block of 20 trials was presented before each task and for each experiment. For the target task, training trials were played in a decreasing order with respect to the protection zones and for the masker task, the order was increasing. Participants verbally indicated the existence of the deviants and the correct answer was given afterwards by the investigator.

In the MEG experiment C, 3 identical blocks of 72 trials (3 protection regions ×4 conditions ×6 exemplars) presented for each task (totaling 432 trials), whereas in MEG experiment D, only the 8 semitones protection zone stimuli was used and more trials from the same condition were collected. 3 identical blocks of 60 stimuli (1 protection region ×4 conditions ×15 exemplars) were presented for each task (totaling 360 trials). For both parts, the inter-trial intervals were randomly chosen to be 1.8, 1.9, and 2.0 s. Participants were allowed to rest after each block, but otherwise required to stay still. For both target and masker tasks, an identical stimulus ensemble (including identical inter-trial intervals was presented for all subjects and the participants were asked to listen for the presence of a frequency deviant in the target rhythm (target task), or duration deviant in the masker (masker task), based on the task order. Each task deviant was present in exactly half of the trials.

In the main experiment, participants were presented with three blocks of stimuli described above. They performed both the masker and the target tasks, with task orders counterbalanced across participants, and were instructed to press a button whenever they heard the appropriate deviant. The button controller was held in the right hand, far away from the sensors. Each stimulus was presented only once, and no feedback was given after each trial. The entire session of both tasks lasted approximately 2 hr.

### Data Analysis

#### Behavioral performance analysis

To evaluate the ability of the participants to perform each task, a d-prime (d′) measure of performance was calculated [22]. The hit rate and false alarm probabilities corresponding to deviant detection for each requested task were calculated and converted to z-scores to compute the d′ value.

To investigate the effect of the pure tone frequency of the target sequences on the behavioral responses in psychoacoustic experiment A, the stimuli were divided into two spectral groups (*low-frequency* target and *high-frequency* target), depending on whether the target tone was lower or higher than the middle frequency 353 Hz (those with target frequency of 353 Hz itself were randomly assigned to low- or high-frequency classes). Then we derived a d′ measure for each frequency class and across different tasks.

To study the build-up of detectability of the target deviant in psychoacoustic part A, we divided the deviant trials into 5 groups according to the deviant's location in time, such that each group covered two possible temporal locations for the deviant throughout the stimulus sequence (out of 10 possible temporal locations for deviants). The hit probability was measured for each group and the false alarm rate was averaged over all groups, independent of its occurrence time because of the uncertainty in false alarm trials. The specific hit rate for each time segment and the averaged false alarm were used to calculate the d′ value for corresponding segments. Only one participant had a strongly negative d′ (−0.7) due to a high false alarm rate and low hit rate, and was excluded from the analysis of build-up.

**Figure 2:**
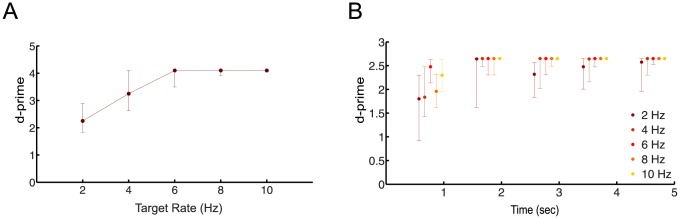
Behavioral performance improvement with target sequence rate reflected in neural build-up curve. (A) Behavioral performance (Psychoacoustic experiment B, N = 12) as a function of target sequence rate for an expanded range from 2 to 10 Hz, in steps of 2 Hz. Overall performance increased with presentation rate, eventually reaching the ceiling value of d' = 4.1 (for 200 trials) (B) Build-up of the behavioral performance as a function of presentation rate. The time for achieving ceiling detection performance is reduced for faster presentation rates. Results are depicted as median and [25,75]% percentiles.

For the psychoacoustic experiment B, d′ values were computed as a function of different target sequence rates (Fig. 2A). We repeated the same build-up analysis as above for different rate conditions to investigate the interaction between target sequence rate and build-up of target detectability (Fig. 2B).

#### Neural Data analysis

To analyze recordings from MEG experiments C and D, in each trial the temporal range from 1.21 to 5.5 s was selected to exclude onset effects. All shortened responses were concatenated then to make an extended response with duration *T* = 4.29 s× number of trials × number of blocks, for each channel and for each task block. Each extended response was translated to the frequency domain using a discrete Fourier transform (DFT), yielding a frequency spectrum from 0 to 500 Hz at a resolution of 1/*T* Hz. The complex magnetic field strength was obtained by the product of the DFT and the sampling interval (1/*f_s_*). Power spectral densities were computed by squaring the complex magnetic field strength and normalizing by the duration *T* of the signal. Then we calculated the square magnitude of the frequency component at 7 Hz, divided by the average square magnitude of the frequency components in a window around 7 Hz (1 Hz on each side), excluding the component at 7 Hz. The resulting quantity will be referred to as the *Normalized Neural Response at 7 Hz* and we averaged this quantity over the 20 channels with the strongest normalized responses for each participant. For channel selection, we pooled all trials together regardless of the performed task and 20 best channels with strongest response to the target sequence were chosen. The average square magnitude of the frequency components in the mentioned window (excluding the 7Hz frequency bin) did not show any significant difference across tasks, so the normalization is task independent i.e. it was not biased by one of the two tasks.

To explore the effect of protection zone width on neural response strength, normalized response amplitude for target and masker tasks per protection zone width were calculated for each participant and averaged over all 12 participants in MEG experiment C.

We investigated the effect of attention on neural response strength by taking the ratio between the normalized responses to the target vs. masker tasks per participant in MEG experiment D.

As described above, the effect of the pure tone frequency of the target sequences on the behavioral responses in MEG experiment D was explored by dividing the stimuli into two spectral groups (*low-frequency* target and *high-frequency* target) depending on whether the target tone was lower or higher than the middle frequency 353 Hz. Then the normalized neural response was calculated for each frequency class and across different tasks.

To study the effect of attention across different frequencies in MEG experiment D, the difference of normalized responses for two tasks was calculated at 7 Hz and 5 other frequencies: two adjacent bins (7 Hz - *df* and 7 Hz+*df*), with *df* = 7/30 Hz and 3 other frequencies in theta, alpha and low beta frequency bands that were multiple integers of *df* (21 *df*≈4.9, 43 *df*≈10 and 64 *df*≈15). Calculated differences did not show a significant task-dependent effect, since there is not a significant difference over average squared magnitude of the frequency components between 6 Hz and 8 Hz except for the 7 Hz.

A sensor-based coherence analysis analogous to that in [12] was performed. Since the results were so similar to those of the earlier study (only the target rate shows a significant enhancement [signed ranks test, p<0.001], both within and across hemispheres), they are not reported.

To analyze the possibility of hemispheric difference in response to stimuli in MEG experiment D, the 20 best channels i.e. with the strongest normalized neural response at the target sequence rate, were chosen from each hemisphere separately. The hemispheric normalized neural responses showed no significant lateralization in either task, in contrast to analogous results with the present paradigm at 4 Hz [12], hence hemispheric differences were not further analyzed. The significance of this lack of lateralization is addressed in Discussion.

The build-up of detectability was studied in MEG experiment D by dividing the entire responses into five temporal segments of approximately 714 ms duration since shorter segments did not show any buildup effect. Corresponding segments extracted from all trials were concatenated to form single extended responses with duration T≈0.714 s×60 trials ×3 blocks for each channel. Then we computed discrete Fourier transform (DFT) from each single response, resulting in a single Fourier response in the range from 0 to 500 Hz with a frequency resolution of 1/T Hz. Different segment durations were used to find the time-scale on which the build-up can be best resolved. Segment lengths were chosen to span an integer number of periods at 7 Hz since we expect to see the build-up in detectability over time windows corresponding to the target sequence rate of 7 Hz.

#### Behavioral versus neural correlation and bootstrap analysis

We correlated the effect of high versus low target frequencies on the behavioral and normalized neural responses from MEG experiment B by correlating the psychometric and neurometric measures for each subject. Specifically, we computed



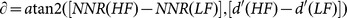



Where NNR(HF) and NNR(LF) stand for averaged normalized neural response at High (HF) and low frequencies (LF). This slope angle represents the relationship between the effects of target frequency and neurometric/psychometric measures. The reason for the use of slope angle rather than slope is that bootstrap analysis produced occasional instances of infinite (and zero) slope, whereas converting the slopes into angles removed this mathematical inconvenience. It also maintains the virtue of keeping within-subject correlations including their sign relation, but discarding absolute co-scaling of the two measures. The across-participant angle was then combined using circular statistics to yield an angular mean for each task [23]. As a preprocessing step, we scaled the neural data (the normalized responses to target) by a factor of two in order to match the absolute ranges of both neural and behavioral values.

We then performed a bootstrap procedure in order to confirm the positive (respectively, negative) correlation between the neurometric and psychometric functions in the target, respectively, masker task. We followed a balanced bootstrap sampling procedure [24], by randomly selecting 12 participants with replacement and computing their angular sample mean and repeating this process 1000 times. The procedure was controlled to ensure that all participants appeared the same number of times over all 1000 bootstrap samplings. Confidence measures were then derived from the bootstrap statistics.

#### Neural source localization

In order to localize the source regions in the brain underlying the magnetic response in all MEG experiments, we used equivalent current dipole analysis. A limited set of complex equivalent current dipoles, best fitting the complex magnetic field configuration at 7 Hz peak in each hemisphere were computed [25]. Only cortical sources are considered since MEG is not sensitive to subcortical neural sources. The same localization process was done for the M100 neural responses obtained in an auditory test prior to the experiment, in which pure 1 kHz tones was presented to the subjects. Significance of the relative displacement between the target and M100 dipole sources were determined by a two-tailed paired t-test in each of three dimensions: lateral/medial, anterior/posterior, and superior/inferior. The Goodness of Fit was computed as the residual variance ratio, as a function of the complex current-equivalent dipole [25]. Only channels with SNR >4 were used in the fitting.

#### Statistical Analysis

Non-parametric tests were used throughout the study to avoid assumptions regarding distributional shape. Single group medians were assessed with the Wilcoxon signed rank test, two group median comparisons with the Mann-Whitney U-test, and multiple groups with the Friedman test, all available in the Matlab Statistics Toolbox (The MathWorks, Natick).

## Results

### Psychoacoustic Results

#### Wider protection zones facilitate the target task and increase build-up speed

The protection zone—i.e., the spectral energy gap around the target sequence—partially controls thedifficulty of segregating the target from the competing maskers background and can induce varying degrees of stream formation.

We investigated the effect of protection zones ranging from 0 to 16 semitones in steps of 4 semitones in psychoacoustic experiment part A (Fig. 1B), asking participants to perform the target task. A positive correlation between the protection zone width and behavioral performance of the target task was measured using bootstrap across participants (p<0.001). An exponential recovery curve was fitted to the performance curve, yielding a decay constant of 9.2 semitones and a positive asymptote of 4.4 starting at 0.8. This indicates a progression of the behavioral performance over a large range of protection zones. Notably, even with no protection zone (PZ = 0), performance remained above chance (d-prime  = 0.8, signed ranks test, p<0.001, d-prime value for chance level is 0), since in this case a frequency change in the target sequence was cued by a disappearance of the target tone at its expected frequency.

We next investigated the build-up of streaming by considering the progression of behavioral performance when the deviants were placed at different times in the target sequence. In the target task the detection performance followed roughly an exponential time course and improved with the width of the protection zone (Fig. 1C, inset shows data from a sample subject to reveal the trend for buildup speed as a function of protection zone). This is quantified by the asymptotic values of the exponential fits being positive, a necessary condition to demonstrate build-up (Bootstrap across participants, p<0.001).

Time constants of the fitted exponentials decreased significantly from 0 semitones to 4 semitones (6.2 to 5.1 s, bootstrap across participants, p<0.001) and from 4 semitones to 8 semitones (5.1 to 2.6 s, bootstrap across participants, p<0.001), but did not change significantly from 8 to 12 semitones (2.6 to 2.4 s, p>0.05). It also had a significant drop from 12 semitones to 16 semitones (2.4 to 1 s, bootstrap across participants, p<0.001). To better demonstrate the distribution of time constants as a function of different protection zones, a histogram of the time constants for fitted exponential curves to the behavioral buildup curves of individual subjects is plotted for different protection zone widths in Fig. 1C. The inset shows example buildup curves of an individual subject. These results suggested that the detection of the target task was easier for larger protection zones, while more buildup time was required for smaller protection zones.

#### Faster presentation rates facilitate the target task

Facilitation of task performance in the context of stream segregation has been studied in a number of previous studies [4], [26], [27]. Here, we studied the effect of the presentation rate of a sequence of stimuli for its known influence on stream formation in the well-known ABA two-tone paradigm [15]. We investigated this dependence in the Informational Masking stimulus tasks with targets at different rates in psychoacoustic experiment B (Fig. 2A). Using a fixed protection zone width (8 semitones), the rate was varied between 2 and 10 Hz in steps of 2 Hz. The trials were presented in 5 consecutive blocks corresponding to 5 different rates. Over the range of tested rates, the performance showed significant variation, with higher rates leading to improved detection performance (Fig. 2A, signed rank test, p<0.0001). Behavioral performance increased over 2 and 4 Hz presentation rates and hit the maximum level at 6, 8 and 10 Hz (Fig. 2A). Looking at the build-up of task detectability as a function of presentation rate, faster build-up is observed for higher presentation rates (Fig. 2B, d-prime  = 2.65, for the 40 trials in each condition).

### *MEG* Results

#### Magnetic field distribution showed a stereotypical pattern for neural activity

The magnetic field distributions of the target sequence rate response component revealed the stereotypical pattern for neural activity originating separately in the left and right auditory cortex. The neural sources of all target rhythm response components with high signal-to-noise ratio (SNR>4) originated in auditory cortex [25]. The mean displacement of the neural source from the source of the auditory M100 response [28] was calculated for each hemisphere. The displacement was significantly different in the anterior direction for both right (11.5±5.8) and left hemisphere (10.8±4.3), using a two-tailed t-test (t = 3.1, p = 0.022 in the right and t = 2.4, p = 0.016 in the left hemisphere), but no statistically significant displacement was observed in other directions.

Goodness of fit for these sources was 0.6±0.18 (artificially reduced in accordance with [25]). Assuming a M100 origin of planum temporale, this is consistent with an origin for the neural response to the target rhythm in Heschl's gyrus, the site of the core auditory cortex, a region known for its good phase-locking to most naturally occurring rates (<40 Hz) [14], [29], [30].

#### Attentional modulation of response power and phase coherence

Neural responses to the acoustic stimuli are expected to reflect the physical attributes of the stimulus, but also aspects of the subject's attentional state. Since the stimuli were acoustically identical for the two tasks, differences in the neural responses during the two tasks must arise only from differences in the attentional state.

**Figure 3:**
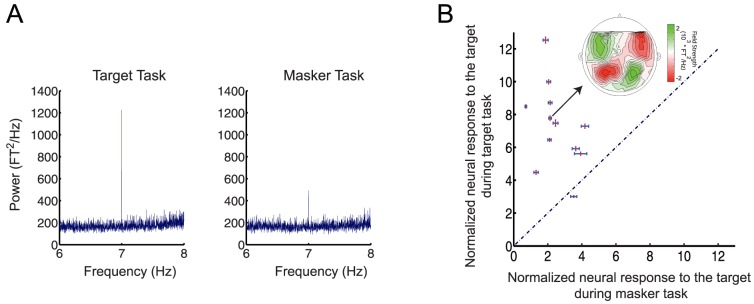
Attention modulates the normalized neural response. (A) The power at the target sequence rate is larger in the target task compared to the masker task (MEG experiment D, N = 12, 20 best channels selected for each participant, see Methods for details). (B) Normalized neural response to the target sequence for each participant is plotted in target-masker normalized response space for each participant. The normalized neural response is computed as the ratio of the neural response power at the target sequence rate (7 Hz) to the average power of the background neural activity (from 6–8 Hz). Error bars represent the standard error for the target task (red, orthogonal bars) and the masker task (blue, horizontal bars). Inset: the MEG magnetic field distributions of the target rhythm response component for a single participant, with red and green representing the target magnetic field strength projected onto a line with constant phase.

We used the phase-locked response to the target sequence rate at 7 Hz in MEG experiment C, as an indicator for the strength of representation of the target stream [12]. As expected, this phase-locked response was stronger during the target task than during the masker task, as indicated by the amplitude of the response power spectrum at 7 Hz (Fig. 3A).

The individually normalized neural responses at a target rate of 7 Hz showed a larger average power gain than for 4 Hz [12]. Power gain is here defined as the ratio of normalized neural response to the target sequence in target vs. masker task. For the present case of 7 Hz the power gain was 3.86 (SEM = 0.87, Fig. 3B, red error bars  =  target task, blue error bars  =  masker tasks), compared to 2.1 at 4 Hz in [12]; additionally, overall amplitudes at 7 Hz were almost a factor 10 smaller than those at 4 Hz. This overall reduction in amplitudes was likely a consequence of the well-known low-pass property of auditory cortical responses [31]–[34].

#### Wider protection zones facilitate the target task, but not the masker task

**Figure 4:**
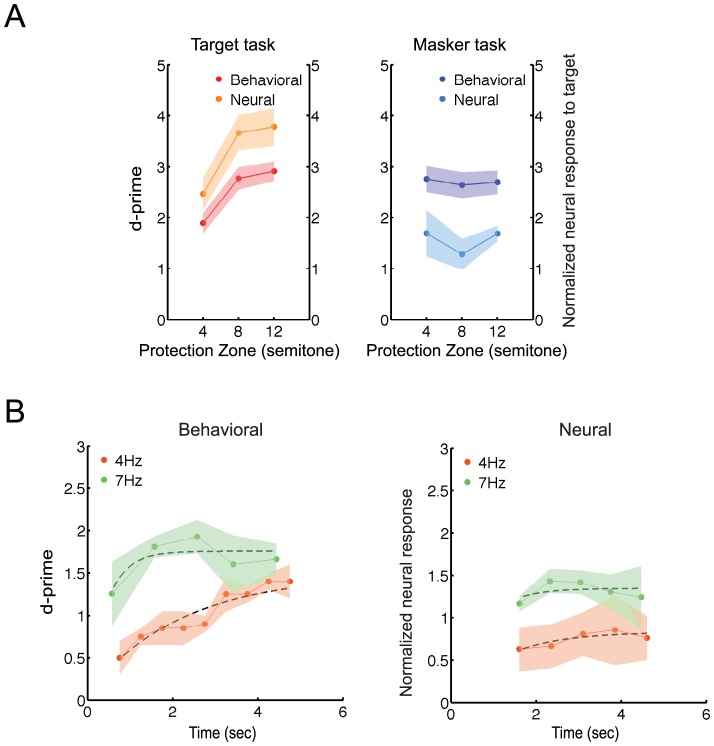
Larger protection zones ease the target task, but not the masker task. (A) Behavioral performance and neural results (MEG experiment C, N = 12) for the target task (left panel) and the masker task (right panel), as a function of protection zone. (B) Analysis of neural and behavioral build-up over time for the target task. Behavioral performance (left panel) and normalized neural responses, normalized with respect to the masker task neural response power (right panel) are plotted as a function of time for both the 4 and 7 Hz target sequence rate (orange and green curves, respectively), averaged over participants. Data shown for the 4 Hz target rate is obtained from the study by Elhilali et al. 2009. Neural responses and corresponding behavioral performances are acquired only for the 8 semitones protection zone.

To get a better understanding of the neural mechanism underlying performance increase as a function of wider protection zones as shown above, we used 4, 8, and 12 semitone protection zones to perform MEG experiment C. Behavioral and neural results for both target and masker tasks are shown in Fig. 4.

For the target task, widening the protection zone facilitated the segregation of the target tones and hence the detection of the frequency deviant (Fig. 4A, right panel, signed rank test, p<0.001; significantly positive slope, bootstrap across participants, p<0.001) in agreement with the results obtained in psychoacoustic experiment A. A corresponding increase in the normalized neural response to the target sequence as a function of protection zone was consistent with the changes in the behavioral results (Fig. 4A, right panel, signed rank test, p<0.001; significantly positive slope, bootstrap across participants, p<0.001). However, for the masker task, increasing the protection zone did not have a significant effect on behavioral performance (Fig. 4A, left panel, signed rank test, p = 0.21). Consistently, there was no significant change in neural activity recorded during the same task (signed rank test, p = 0.1).

#### Faster rates facilitates the buildup of target detectability

As an extension to psychoacoustic study part B, we examined whether the normalized neural responses reflected a similarly rapid build-up of performance for higher target presentation rates To this end, neural and behavioral responses from MEG experiment D were compared with those of a 4 Hz target rate [12]. In the current study, behavioral detectability of the target deviant was calculated for each of the 5 time segments corresponding to the target deviant's location (Fig. 4B, left panel, green). The build-up of the normalized neural response was measured over the duration of the trial by separating the response into non-overlapping segments and computing the 7 Hz contributions in each segment. No build-up was observed for window sizes less than 5 cycles, likely due to lack of sufficient statistical power. A weak build-up as a flattened curve was obtained for segment length approximately 714 ms (5 cycles) (Fig. 4B, right panel, green), consistent with the progression speed of behavioral response (in MEG recording session, left panel, green). Time constants given by fitted exponentials to both neural and behavioral curves in the 7 Hz target, were significantly positive but small (0.63 s for neural curve and 0.1 s for behavioral curve, signed rank test, p = 0.03). Given the fast build-up obtained psychoacoustically for the 8 semitone protection zone of the 7 Hz target (Fig. 1C, 3rd panel), we conjecture that a fast neural build-up was occurring at the beginning of trials, but early enough that it could not be resolved using the current analysis.

To further validate this analysis, we reanalyzed the old 4 Hz target data from [12]. For better comparison of the neural build-up curves, the normalized neural responses for both 4 and 7 Hz target sequence rates were further normalized by the average power of the normalized neural responses in the corresponding masker tasks (Fig. 4B, right panel). A significant build-up was obtained using a 750 ms time windows (three periods) for 4 Hz. The time constants obtained from exponential curve fittings were significantly positive (1.17 ms for the neural data curve and 11.8 ms for the behavioral data, signed rank test, p<0.01) and larger than the ones for the 7 Hz curves (rank sum test; p<0.003 for behavioral curves and p<0.02 for neural curves), suggesting that the detection task for the 4 Hz target sequence embedded in a 8 semitone protection region was harder than the detection task for the 7 Hz target sequence under similar conditions.

#### High frequency targets facilitate the target task

**Figure 5:**
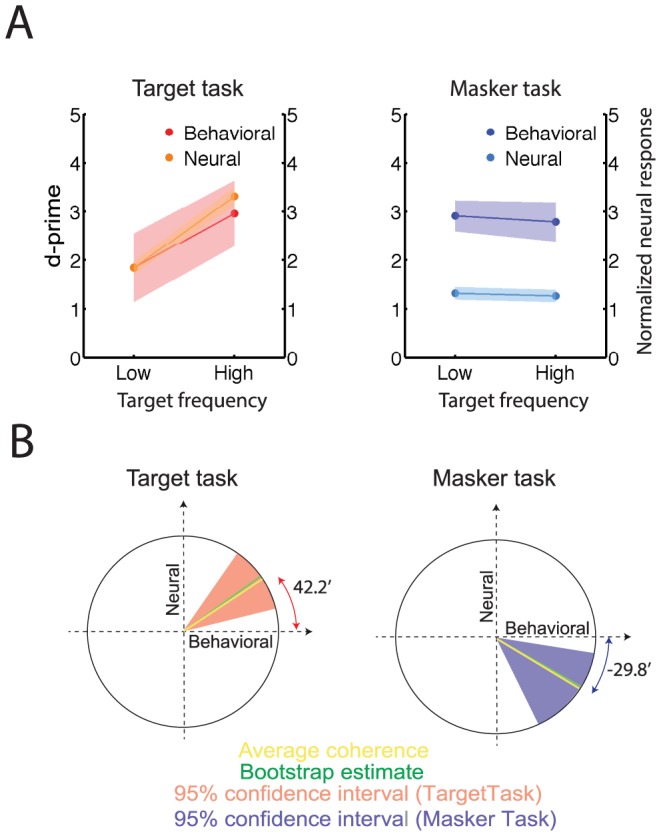
Bottom-up saliency of the target sequence increases for higher target frequencies. (A) Behavioral and neural responses (MEG experiment C, N = 12) as a function of target frequency. In the left panel, the red/orange line corresponds to behavioral and neural responses for the target task with respect to the low and high target frequency. In the left panel dark/light blue corresponds to behavioral and neural responses for the masker task. Error hull represent 1 SEM. (B) Correlation of the behavioral and neural responses as a function of target frequency. The ratio of the neural to behavioral response differences as a function of target frequency is averaged across participants. A mean slope angle of 42.4° for target (left plot) task and −29.8° for masker (right plot) task (yellow line) were obtained in this analysis. As detailed in Methods, the slope angle corresponds to the strength of correlation between neural and behavioral data. Bootstrap estimates (overlying green lines) and their 95% confidence intervals (pink and blue background for the target and masker task, respectively) confirm the positive/negative correlations for target/masker task.

Acoustic stimulus parameters influence the saliency of a streaming percept. One such parameter is the frequency of the target tone sequence, which influenced the results both for behavioral and neural data. In MEG experiment D, target sequences were divided into high and low frequency tones (above or below 353 Hz). Both behavioral and neural data showed a significantly positive slope (bootstrap across participants, p<0.001) as a function of target frequency in the target detection task (dark/light red line, Fig. 5A, left panel), indicating that high frequency tones facilitated target detection. Neither slope was significantly non-zero in the masker task for the average behavioral and normalized neural responses (Fig. 5A; right panel); however, the individual behavioral and normalized neural response trends showed a significantly negative correlation as explained below.

To better demonstrate the correspondence between the normalized neural response and behavioral measures, we computed the correlation between the two indicators during both tasks as a function of target frequency. As described in Methods, we computed an angular measure relating neurometric and psychometric changes as a function of frequency. The resulting average angle over subjects was positive 42.4° for the target task and −29.8° for the masker task (Fig. 5B, yellow line). Bootstrap analysis was performed across participants and the estimated angle is plotted as a green line with the corresponding 95% confidence interval as the pink/blue backgrounds for the target/masker tasks. The positive and negative correlations obtained for target and masker task respectively, confirmed that behavioral performance in the target task is better for higher frequency targets (>350 Hz) than for lower frequencies (sum rank test, p<0.01). An increase to the neural response of the target is correlated with this trend. Conversely, the masker task showed a trend of being oppositely affected by the physical saliency of the target task despite the independence of the two tasks.

#### Attention to the target stream leads to selective power and phase enhancement at target rate

**Figure 6:**
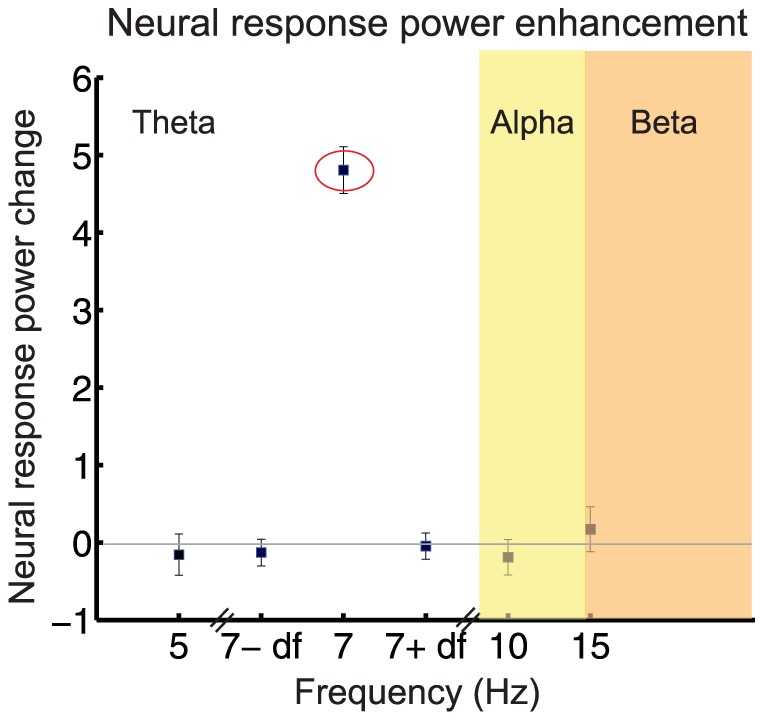
Stream formation recruits more widespread brain areas at the target sequence rate. Power enhancement during the target task. The difference between the normalized neural responses in the target task versus the masker task (MEG experiment D, N = 12) shows a significant and highly precise enhancement at the frequency of the target sequence (7 Hz, circled in red). Error bars represent 1 SEM in each graph.

The normalized neural response to the 7 Hz rhythms obtained from MEG experiment D, showed a significant increase in the target vs. masker task (Fig. 6, signed rank test, p<0.0001). In contrast, no significant change in the normalized neural response to the nearby or distant frequencies was obtained, suggesting that the sustained attention to the target stream leads to a feature-selective modulation of the cortical response, but has no significant impact on responses to the other nearby or distant frequencies (signed rank test, p-values  = 0.15).

## Discussion

Stream formation is a central process in parsing out the acoustic environment. Perceptual cues and attentional focus modify this segmentation, both qualitatively and quantitatively.

In the present study, we pursued two main goals. The first goal was to examine the correspondence between the mechanisms and percepts of stream segregation versus those of the informational masking paradigm, and especially their dependence on the spectrotemporal properties of the stimuli. The second goal was to investigate the potential mechanisms of stream segregation and their interaction with selective attention, for which we employed neuromagnetic imaging. Specifically, while holding the stimulus fixed, we investigated the changes in the neural responses as attention was directed to different components of the acoustic scene. This was repeated under different spectrotemporal stimulus conditions so as to explain the integration of perceptual features of a complex acoustic scene mediated by the processes of attention.

We based our experimental paradigm and analysis on the hypothesis that target sequence detection relies on similar neural mechanisms both in our informational masking paradigm and the classic streaming paradigm.

This hypothesis is supported by a number of earlier studies in which the similarities between these paradigms were discussed [8], [12]. First, in line with the arguments discussed in [8] , we hypothesize that systematic dependence of performance on the size of the protection zone is analogous to the frequency separation parameter in streaming experiments using the classic two tone paradigm [1]. This dependence ultimately relies on the frequency selectivity of neurons in the central auditory system, a low-level neural mechanism [10], [18]. It was also shown in [8], that detection performance in the target task decreases if the target tone is presented every other burst. This is consistent with results in [35], that the degree of streaming is related to the gaps between successive tones in a stream. Moreover, in regard to buildup, it has been shown in the classic streaming paradigm that detection improves in the target task with increasing the number of tone bursts in the target sequence, quite similar to the analogous effect here. The underlying mechanism in both cases might be explained via accumulation of sensory evidence, with a causal relationship to the build-up of stream segregation.

While these arguments rule out some potential differences between the IM and two-tone cases, it is also possible that detection of the repeating target tones could have different mechanisms on different levels, and their effects nonetheless are correlated on the behavioral level.

Comparing behavioral and neural measures, we have confirmed that attending to one stream significantly modulates the neural response to the attended stimulus. Despite the known transient effects of attention on auditory signals [36], [37], a sustained increase in the normalized neural response was found to correlate with sustained attention. This enhancement is consistent with the behavioral improvement in target detection for individual subjects, which supports the hypothesis that attentional manipulation can lead to increased responses to the attended features, and suppression of the response to the background or unattended features [12], [38]–[43].

It is also possible that the neural response enhancement in the target task arises directly from the occurrence of more trials in which listeners are aware of the presence of the target sequence vs. masker task. Therefore, this study cannot directly establish whether the enhanced neural power is a cause or an effect of selective attention.

Recent studies suggest that oscillatory entrainment in various frequency bands can be enhanced by attention [44]–[46]; however, the results of this experiment are unlikely to be a consequence of entrainment since the normalized neural responses show a significant power change only at frequency of the target presentation rate and no other frequencies, even nearby frequency bins (See results and Fig. 6).

We also observed a systematic dependence of performance and normalized neural response strength on the width of protection zone for the target task. This is analogous to the increase in frequency separation between two-tone sequences studied in the more traditional A-B-A streaming paradigms [1]. According to our findings, increasing the spectral separation improves behavioral detection and, notably, also causes an increase in the normalized neural response. This can be speculatively attributed to well-known lateral inhibitory interactions, which may occur between tones as much as an octave or more apart in cortex [47]–[49]. In this case, the boundary between energetic and informational masking becomes somewhat blurred, and very close spectral distances between target and masker tones eventually inhabit the same frequency band (dependent on the tuning width of auditory neurons), and thus could be considered as energetic masking rather than informational masking. But it is also possible that these suppressive interactions between the two temporally incoherent streams (target and masker) are inherently due to the desynchronized activation of these respective frequency channels, and not simply to pre-existing inhibitory connections [50]. If so, we would predict that this enhancement of response amplitude with increasing frequency separation would also occur for two alternating tones despite the fact that they are not simultaneous. Given our observation of no significant change of the neural response for the masker task, we postulate attention to be a required component to instantiate the increase of neural responses in the target task.

Higher target tone frequencies produced stronger normalized neural responses, and their deviants were easier to detect. This effect may be due to an enhanced bottom-up saliency that increases as a function of frequency, i.e. tones at higher frequencies (350 Hz to 500 Hz) are perceived to be louder compared to low frequency tones (250 Hz to 350 Hz) at the same amplitude (ISO 226∶2003). Interestingly, the strong, *positive* correlation between the behavioral and normalized neural response for the target task was complemented by a significant *negative* correlation in the masker condition. Thus, subjects with a positive/negative behavioral trend as a function of sound frequency, showed a decrease/increase in their corresponding normalized neural response, respectively. This could be explained by the competitive nature of the tasks, i.e. better detection in the masker task requires more effective suppression (decrease) of the competing target sequence. This finding is also significant as it confirms that the difficulty of the tasks was sufficient to manipulate the listener's attention towards or away from the target sequence.

Similarly, increasing the presentation rate of the target sequence had a significant, positive effect on behavioral performance (2–10 Hz range) and its neural correlates in the target detection task (4 vs. 7 Hz), presumably because of the more rapid buildup of target/masker segregation (streaming). This is consistent with previously measured effects of temporal rates in auditory scene analysis in which faster rates induced stronger streaming effects [1]. An earlier study by Xiang et al. 2010 [16] found conflicting results when presenting competing pairs of different temporal rate sequences (4 and 7 Hz) to the subjects, and instructed them to attend to one of the two rhythms and detect in it a deviant temporal jitter. This psychoacoustic study found a streaming advantage for the 4 Hz rhythms relative to the 7 Hz, inconsistent with our current findings and classical streaming studies [1]. We conjecture that this may simply have been a consequence of the reliance on temporal jitter as the deviant, which is more difficult to detect with faster rates, leading to a decrease in the detection scores of the 7 Hz sequence.

Finally, average temporal alignment (termed ‘coherence’) has recently been suggested as a dominant contributor to stream formation [12], [50], [51]. According to the temporal coherence hypothesis, distinct neural populations with temporally correlated responses are grouped together representing one single stream, whereas neural populations with uncorrelated temporal responses are segregated representing different streams. In the present study, temporal alignment did not play an important role for binding across multiple frequency channels, since target and masker streams were temporally uncorrelated (due to the random nature of the masker). However, this lack of coherence may have been used as a discriminating factor, strengthening the perceptual and neural activity independence between the target and masker components of the stimulus, leading to a better target/masker segregation ability for listeners.

There was no measurable hemispheric asymmetry in either task. This is in contrast to the left-biased hemispheric asymmetry seen for a 4 Hz target rate during the target task in [12] (not the 7 Hz of this study). This may be due to a cancellation of competing asymmetries, since a right-biased asymmetry has also been observed for 7 Hz (during a different task than in this study; different competing stimulus and different deviants than in this study, but the targets themselves were identical) [16].

Using both behavioral and neural measures we have shown that conditions, which facilitate target detection, are paralleled by enhancements in neural activity. This suggests that the neural sources of the MEG signal associated with the target sequence are already affected by the conditions that give rise to the streaming percepts, and are in fact good indicators of the perceptual state of the subjects in perceiving the presence of informational masking in the auditory scene, and perhaps other scenes as well.

### Conclusion

Stream segregation/formation can be performed effortlessly by human subjects in many natural scenes; for example, in crowded environments, we are able to listen to a specific speaker and perceive the attended speech as a distinct auditory stream, despite the complex acoustic signal we receive from all other sources such as other speakers and music. The present study indicates that different properties such as bottom-up saliency of target frequency, top-down attentional modulation and frequency separation in an auditory scene, influence our streaming ability significantly, suggesting that they correspond to properties that distinguish the emitters of different sounds.

Further, the present findings shed some light on the similarities and differences of the modulation rates in the range of 2–10 Hz, which are known to be crucially important in grouping the physical and perceptual cues in a complex acoustic scene and stream formation [13]–[15].
